# Dairy Cow Behavior Is Affected by Period, Time of Day and Housing

**DOI:** 10.3390/ani12040512

**Published:** 2022-02-18

**Authors:** Lisette M. C. Leliveld, Elisabetta Riva, Gabriele Mattachini, Alberto Finzi, Daniela Lovarelli, Giorgio Provolo

**Affiliations:** 1 Department of Agricultural and Environmental Sciences, Università degli Studi di Milano, 20133 Milan, Italy; elisabetta.riva@unimi.it (E.R.); mxlelex@libero.it (G.M.); alberto.finzi@unimi.it (A.F.); giorgio.provolo@unimi.it (G.P.); 2 Department of Environmental Science and Policy, Università degli Studi di Milano, 20133 Milan, Italy; daniela.lovarelli@unimi.it

**Keywords:** dairy cattle, heat stress, accelerometers, barn microclimate

## Abstract

**Simple Summary:**

Many factors, such as the climate, period of the year, time of day and housing, are known to affect cow behavior. However, it is not yet clear what is the combined effect of these factors. For instance, it is unclear whether warmer weather only alters cow behavior during the day or also during the night. Therefore, a survey was performed on eight dairy cow farms in Northern Italy in three periods: summer, winter and a temperate season (spring or autumn). Sensors were installed to monitor the temperature and humidity. Cow behavior was monitored with cameras and with accelerometers that were placed on their legs. These methods allow us to determine how much time the cows spent lying, standing or feeding. We found that both daytime and nighttime behavior differed between the periods and that housing had an effect not only on the behavior itself but also on how it changed between the periods and from daytime to nighttime. These findings show the importance of measuring behavior during both daytime and nighttime and illustrate the influence of the barn structure and farm management on cow behavior and welfare.

**Abstract:**

Dairy cow behavior is affected by external and endogenous factors, including time of year, barn microclimate, time of day and housing. However, little is known about the combined effects of these factors. Data were collected on eight farms in Northern Italy during summer, winter and a temperate season. The temperature-humidity index (THI) was recorded using environmental sensors, whereas cow behavior was monitored using leg accelerometers and cameras. Period, time of day and their interaction all significantly affected lying, standing and feeding behavior. However, although THI had a significant negative effect on lying and a positive effect on standing during daytime (all *p* < 0.001), during nighttime, it only had a significant negative effect on lying duration and mean lying bout duration (*p* < 0.001 for both). There was also significant variation between farms in all behavioral parameters, as well as interactions with period and time of day. For instance, farm differences in lying duration were more pronounced during daytime than during nighttime. These findings show how housing can interact with other factors, such as period of the year and time of day, and illustrate the influence of barn structure and farm management on cow behavior and, consequently, their welfare.

## 1. Introduction

Dairy cow welfare is a long-underrated issue that has recently gained more attention from both scientists and legislators [1,2]. Dairy cows constitute one of the major livestock groups in Europe and the world, and due to their relatively long productive lifespan, their health and wellbeing are not only of ethical but also of economic importance [3]. One of the best ways to study animal welfare is through the observation of their behavior. In dairy cows, it is well established that lying behavior is a useful indicator of their health and welfare (e.g., [4,5]). Sick and lame cows usually increase their daily lying time (e.g., [6,7,8]), while mastitis is often found to reduce lying time (e.g., [9,10]). Heat stress is also often found to reduce lying time (e.g., [11,12,13,14]), as cows prefer to rest while standing to increase the body surface available for cooling [15]. Apart from daily lying time, the number of lying bouts per day and mean lying bout duration are also important parameters to consider since they can be affected by various factors, such as lying surface, cow parity and the wetness of bedding [4]. Usually, when cows change their lying behavior, this also affects other behaviors, in particular standing. As mentioned above, standing increases during heat stress (e.g., [16]) as well as in cows with mastitis [10]. Another important behavioral indicator of cow welfare is feed intake [5]. Reduced feed intake can be both a cause and a consequence of poor health and welfare. Around parturition, cows often reduce their feed intake [17], which, in combination with increased energy demands, could cause metabolic disorders and infectious diseases [8,18]. On the other hand, various conditions, including lameness and heat stress, also lead to reduced feed intake [19,20]. Often, reduced feed intake can be observed days to weeks before a condition is clinically diagnosed [8,21,22]. Although feed intake may be costly to monitor, feeding time has been found to correlate highly with feed intake [23] and, therefore, could be a cost-effective alternative indicator of cow welfare [5].

Commonly, cow behavior is expressed in values that reflect the daily time budget of cows [4,24,25]. This provides a good indication of whether the cow’s nutritional and behavioral needs are met. For instance, cows lie down on average 10–12 h per day, and a reduction in lying time is associated with potential risks to cow welfare, such as increased physiological stress responses and lameness [4]. However, daily patterns in behavior (i.e., the distribution of a behavior during the day) may also be informative of a cow’s welfare status [26,27]. For instance, during periods of heat stress, cows shifted their rumination time towards more nighttime rumination [28], even though absolute rumination time has been reported to decrease during the night [27]. Lying time was reported to decrease in the daytime in summer months and under heat stress conditions [11,16,26,29], whereas nighttime lying was not found to be affected under these circumstances [11,26].

In addition to daily patterns, cow behavior is also known to have seasonal variations (e.g., [26,30]). Several studies have shown that compared to winter and temperate seasons (i.e., spring and autumn), cows lie down much less during the summer months (e.g., [11,30,31]). Feed intake has also been reported to decrease during summer [32,33], whereas standing is reported to increase [30]. These seasonal variations are often ascribed to a combined effect of air temperature and relative humidity, which is quantified by the temperature-humidity index (THI; [31]). Indeed, many studies have found that the THI is a significant determinant of cow behavior (e.g., [11,32]). Above a certain THI threshold (THI > 72 according to [34]; THI > 68 according to [35]), heat stress occurs, which increases the body temperature and induces physiological and behavioral responses, resulting in serious risks to cow welfare and health [35,36]. However, apart from THI, the photoperiod and endogenous circannual rhythms also play an important role in determining farmed animal behavior [37]. Little is known about the effect of the period of the year and of THI on cow daily behavioral patterns.

The type of housing can play an important role in determining cow behavior. For cattle that are housed in barns throughout the year, the barn structure can reduce or increase external climate conditions [38,39]. For instance, higher roofs and proper roof insulation can help to reduce the effects of excessive solar radiation (reviewed by [39]), and the presence of different cooling systems, such as ventilators and sprinklers, can help to increase convective heat loss and increase lying behavior [40,41]. In addition to affecting the barn microclimate, some structural aspects of the barn, such as the size of the barn, stocking density and bedding materials, may also directly affect cow behavior [25,42,43]. For instance, cows have been found to lie down less when the cow/cubicle ratio is less than 1.0–1.2 [4]. Finally, the management of the barn and the cows can also affect cow behavior. Insufficient cleaning can lead to wet and muddy cubicles, which result in reduced lying time [44]. Additionally, more frequent feed delivery and milking were reported to affect the time budgets of dairy cows, reducing the time spent lying and ruminating [45,46,47]. All these factors of housing can interact with variations in behavior due to the period of the year and time of day. However, not much is known about such interactions.

The first aim of this study was to examine the combined effects of the period of the year (winter, temperate seasons, summer) and time of day (daytime vs. nighttime) on cow behavior and to determine the effect of THI on cow behavior for daytime and nighttime separately. We hypothesized that both the period and time of day have significant effects on lying, standing, as well as feeding and that the THI will have a significant effect on behavior only during the day. Specifically, we expect that lying and feeding would be reduced during the warmer periods, mainly during the day, whereas we expect to see an opposite pattern for standing. The second aim of this study was to examine the effect of housing and its interactions with period and time of day on cow behavior. We expected to find that cow behavior varies between the studied farms, which have different housing characteristics, and that the effects of period and time of day differ between farms. Together, these aims will help to better understand cow behavior and to what extent it is affected by period, time of day, THI and housing, allowing a better interpretation of the different types of behavior as indicators of cow welfare.

## 2. Materials and Methods

### 2.1. Study Sites

The data reported here were collected as part of a larger study that was focused on the effect of barn structure on the internal barn climate [48,49]. The data collection took place in eight dairy cattle farms situated in the Po Valley in the northern Italian region of Lombardy. For privacy reasons, the farms are referred to here as A, B, C, D, E, F, G and H (corresponding to the numbers used by [48,49]). Farms were selected based on the location, availability of the farmer to participate in the project and the possibility to install sensors. Data regarding the dairy cows, barn structure and management were collected on the first visit to each farm. In Table 1, an extraction of this data is shown, which is primarily focused on factors that could directly affect cow behavior (for information on other aspects of the farms, please refer to [48,49]). All monitored farms had a loose-housing system with free stalls. In each farm, data collection focused on one group of lactating dairy cows and the entire area in which this group was housed. The monitored cows were all lactating dairy cows belonging to the Italian Holstein breed (mean parity: 2.2, range: 1–9 lactations). On all farms, the cows were fed unifeed, which is a minced mixture of all necessary feeds (e.g., straw, hay, alfalfa, corn silage and minerals). The exact composition differed between farms.

### 2.2. Equipment and Data Collection Procedure

Data were collected for 1 year (2018–2019) in three separate periods of one week each per farm. The three periods took place in different seasons, one during the winter (January and February), one during the summer (August and July) and one during a temperate season (April, May and October). Each farm was visited at the start of each data collection period (day 1) to install all sensors and start the recording. One week later (day 7), the farm was visited again to deinstall the sensors and store the data. Climate data were collected with two sensors (HOBO U12 Temp/RH/Light/External Data Logger: Onset Computer Corporation, Bourne, MA, USA) that were installed at two positions in the monitored area at a height of 2 m. These sensors recorded ambient temperature and relative humidity at 10-min intervals. For behavioral observations, time-lapse cameras (Victure HC200 Wildlife Camera, Govicture, Shenzhen, Guangdong, China) were installed at 3–4 m from the floor (depending on the farm) in the monitored area. The cameras were programmed to take pictures every 10 min and to save these on an SD card. Depending on the size and layout of the monitored area, either two (farms A, B, C, E, G and H) or four (farms D and F) cameras were installed. To monitor the lying behavior in more detail, ten dairy cows per farm were randomly selected from the monitored group and fitted with a HOBO Pendant G Data Logger (Onset Computer Corporation, Pocasset, MA, USA) on the hind leg. Efforts were made to use the same cows across the different periods, but due to lactation and health status, this was not always possible. In the end, 29 cows were monitored in all three periods, 44 cows were monitored in two periods and 60 cows were monitored in only one period. Due to technical problems, data were missing from three cows in winter and two cows in temperate seasons. This device was positioned on the leg with the X-axis perpendicular to the floor and attached with tape and a tough plastic leg band. The accelerometer recorded the degree of vertical tilt of the X and Z-axis at 1-min intervals.

### 2.3. Scan Sampling

The pictures taken by the time-lapse cameras at every full hour for 4 days were analyzed, resulting in 96 pictures analyzed per farm per period. Due to technical problems, pictures were missing for one farm (C) in the temperate season, and only 2 days could be analyzed for farm D in the winter season, resulting in a total of 2160 analyzed pictures. In each picture, the number of cows that were lying, standing or feeding was counted. A cow was counted as “feeding” if it was standing with its head inside the feed bunk. Standing included all cows that were in an upright position and that were not scored as “feeding”. As such, this category includes both standing and walking cows. Lying was defined as lateral or sternal recumbency [50,51]. Because of the use of several cameras and to avoid double counts, virtual lines were drawn to divide the monitored area into two or four sub-areas (depending on the number of cameras). No counts were made during milking, cleaning or other events that affected cow behavior.

### 2.4. Parameter Calculation and Statistical Analyses

Separate analyses were performed for nighttime (22:00–06:59) and daytime (07:00–21:59). These intervals were chosen based on light intensity measurements in the barns during the three periods. At 7:00 and 22:00, the mean light intensity was below 30 Lx (compared to the mean levels in the 50–290 Lx range during the day). Based on the 10-min interval measurements from the two HOBO sensors, means per hour were calculated for the ambient temperature and relative humidity and these means were used to calculate an hourly temperature-humidity index (THI), using the equation suggested by the NRC ([52]; see Table 2). Based on the picture analyses, the cow lying index (CLI), cow standing index (CSI) and cow feeding index (CFI) were calculated to obtain an indication of the proportion of the monitored group that was involved in different activities ([50,51]; Table 2). Means for daytime and nighttime were calculated from all hourly data (THI and picture data). The accelerometer data were used to analyze the cow lying behavior in more detail. The posture of the animal was classified based on the degree of vertical tilt (X-axis) with an axis value of <60°, indicating that the cow was standing, and a value of ≥60°, indicating that the cow was lying down [53,54]. At each change of position from standing to lying, a new lying bout was counted. Based on the accelerometer data, the lying duration (LD), number of lying bouts (NLB) and mean lying bout duration (MLBD) were calculated (Table 2). To control for differences in length between nighttime (9 h) and daytime (15 h) periods, the LD was calculated as a percentage of time, and the MLBD was calculated as number per hour.

The statistical analyses were performed using SAS version 9.4 (SAS Institute Inc., Cary, NC, USA). The effect of the period of the year and time of day on the picture parameters (CLI, CSI and CFI) was tested using a generalized mixed model analysis with the period (winter, temperate and summer), time of day (daytime and nighttime) and period × time of day as fixed factors, day as a random factor and farm as the subject (GLIMMIX procedure; distribution: normal; link function: identity). Pairwise comparisons were made with the Tukey–Kramer test, using the SLICE option to perform a partitioned analysis of the least square means, allowing us to test only relevant comparisons, such as time of day within the same period or periods within the same time of day. To determine if the THI in the barn affected the cows’ behavior differently during daytime and nighttime, separate generalized mixed model analyses were performed per time of day with internal THI as a fixed factor, period and day as random factors and the farm as the subject (GLIMMIX procedure; distribution: normal; link function: identity). Because the accelerometer data were obtained from individual cows within a farm, this also allowed us to additionally test the effect of the farm on the parameters measured with the use of accelerometers (LD, NLB and MLBD). This was tested using a generalized mixed model analysis with the period (winter, temperate and summer), time of day (daytime and nighttime), farm (eight farms), period × time of day, period × farm, time of day × farm and period × time of day × farm as fixed factors, day as a random factor and the cow as the subject (GLIMMIX procedure; distribution: normal; link function: identity). Pairwise comparisons were made with the Tukey–Kramer test, using SLICE options (for the effects of period × time of day and period × time of day × farm) to perform a partitioned analysis of the least square means. Similar to the picture data, separate generalized mixed model analyses were performed per time of day with internal THI and the farm as fixed factors, period and day as random factors and the cow as the subject (GLIMMIX procedure; distribution: normal; link function: identity)

## 3. Results

### 3.1. Picture Data

**Figure 1:**
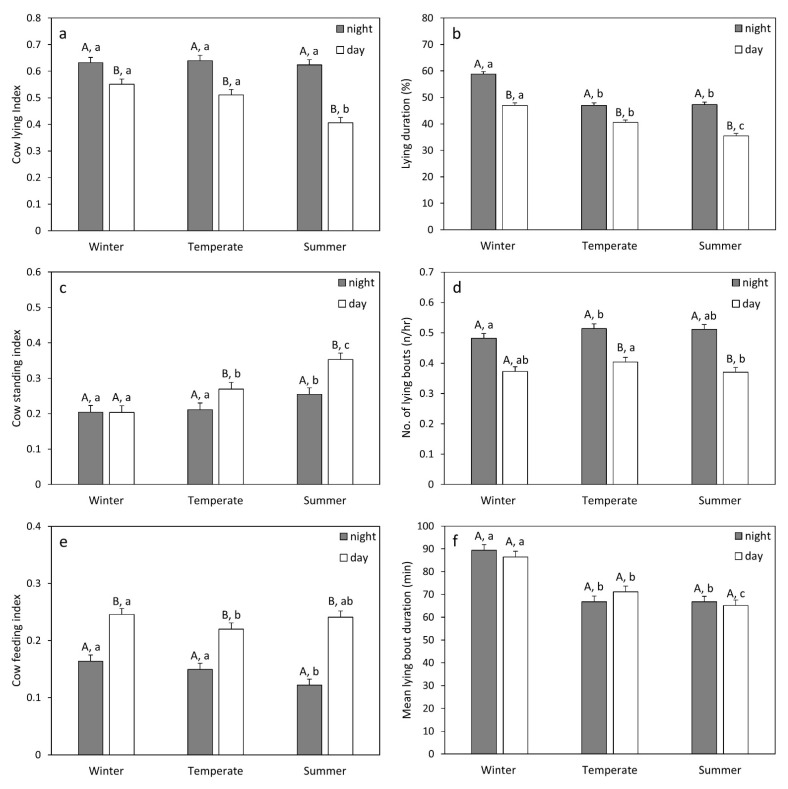
LS means ± standard errors for the cow lying index (**a**), lying duration (**b**), cow standing index (**c**), number of lying bouts (**d**), cow feeding index (**e**) and mean lying bout duration (**f**). The different capital letters (A,B) above the bars indicate significant differences between daytime and nighttime means. The different lower-case letters (a–c) above the bars indicate significant differences between the three seasons (Tukey–Kramer Test; *p* < 0.05).

The period of the year (winter/temperate seasons/summer) and time of day (daytime/nighttime) and their interaction had significant effects on CLI, CSI and CFI (see Table 3 and Figure 1a,c,e). Cows lie more during nighttime than during daytime in any period (winter: t = 4.48, *p* < 0.001; temperate: t = 6.85, *p* < 0.001; summer: t = 12.36, *p* < 0.001). Nighttime CLI was unaffected by period, whereas daytime CLI decreased significantly during the summer compared to winter (t = −0.15, *p* < 0.001) and temperate seasons (t = 0.11, *p* < 0.001). The internal THI had a significant negative effect on CLI during daytime (t = −4.16, *p* < 0.001), but not during nighttime (t = −0.72, *p* = 0.477; Table 4). Cows stand more during daytime compared to nighttime in summer and temperate seasons (temperate: t = −3.57, *p* < 0.001; summer: t = −6.43, *p* < 0.001; Figure 1c), but not in winter (t = 0.03, *p* = 0.978). Daytime standing increased during summer compared to temperate seasons (t = −0.08, *p* < 0.001) and winter (t = 0.15, *p* < 0.001), whereas in the temperate season, cows still stand more than in winter (t = 0.07, *p* < 0.001). Nighttime standing increased in summer compared to temperate seasons (t = −0.04, *p* = 0.018) and winter (t = 0.05, *p* = 0.004). The internal THI had a significant positive effect on CSI during daytime (t = 5.75, *p* < 0.001), but during nighttime, this was only a tendency (t = 2.06, *p* = 0.053; Table 4). CFI is also affected by both period and time of day (Table 3). Cows feed more during daytime in any period (winter: t = −8.89, *p* < 0.001; temperate: t = −7.41, *p* < 0.001; summer: t = −13.38, *p* < 0.001; Figure 1e). Daytime feeding decreased during temperate seasons compared to winter (t = −0.03, *p* = 0.021), whereas daytime feeding in the summer does not significantly differ from other periods. Nighttime feeding, on the other hand, decreased during summer compared to temperate seasons (t = 0.03, *p* = 0.010) and winter (t = −0.04, *p* < 0.001). The internal THI had no significant effects on CFI (day: t = −0.57, *p* = 0.579; night = −1.97, *p* = 0.065; Table 4).

### 3.2. Accelerometer Data

**Figure 2:**
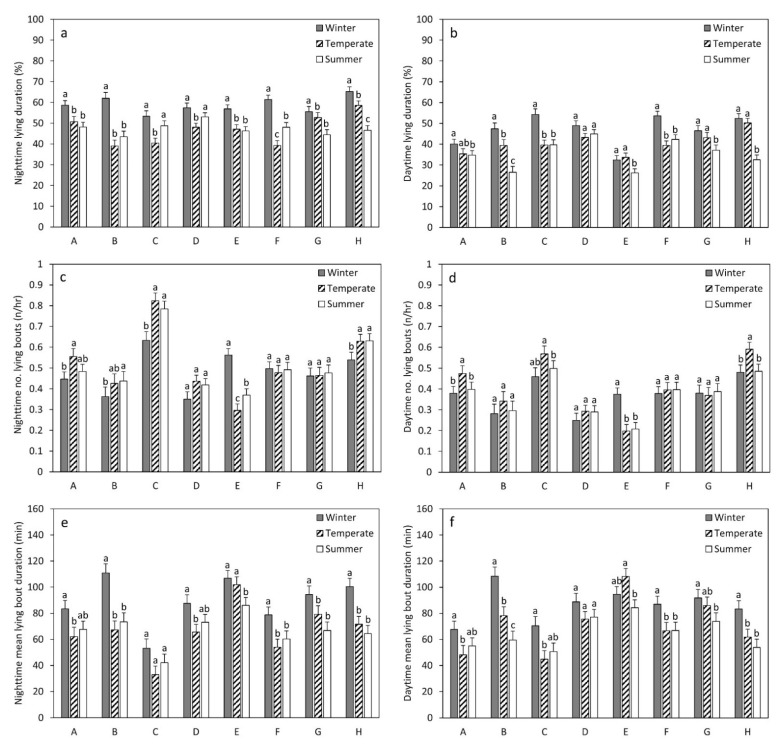
LS means ± standard errors for nighttime lying duration (**a**), daytime lying duration (**b**), nighttime number of lying bouts (**c**), daytime number of lying bouts (**d**), nighttime mean lying bout duration (**e**) and daytime mean lying bout duration (**f**). The different lower case letters (a,b,c) above the bars indicate significant differences between period means per farm (Tukey–Kramer test; *p* < 0.05).

Lying duration (LD) was affected by all included fixed factors, i.e., the period, the time of day, the farm and their interactions (Table 5; Figure 1b and Figure 2a,b). This section will report the results of the analyses with the two SLICE options (the effects of period × time of day and period × time of day × farm). The LD was lower during nighttime than during daytime in all periods (winter: t = 17.49, *p* < 0.001; temperate season: t = 9.59, *p* < 0.001; summer: t = 17.95, *p* < 0.001). During both daytime and nighttime, the LD was significantly lower in temperate seasons and summer compared to winter (daytime: vs. temperate: t = −7.19, *p* < 0.001; vs. summer: t = −12.91, *p* < 0.001; nighttime: vs. temperate t = −13.11, *p* < 0.001; vs. summer: t = −12.89, *p* < 0.001). During the daytime, the LD was also significantly lower during summer compared to temperate seasons (t = 7.30, *p* < 0.001). During the nighttime, there was little significant variation between farms, and the farms also showed quite similar differences in LD between periods (Figure 2a). In contrast, during the daytime, there was more variation between farms. During the winter, the LD was significantly lower on farms A and E than on most other farms. In summer, the LD was significantly lower on farms B and E than on many other farms, whereas it was significantly higher on farms D and F than on many other farms (Supplementary Materials, Table S1). In contrast to all other farms, farm D showed no significant difference between winter and summer in the LD (Supplementary Materials, Table S2). The internal THI had a significant negative effect on the LD, during both daytime and nighttime (day: t = −4.46, *p* < 0.001; night: t = −6.84, *p* < 0.001; Table 4).

The number of lying bouts (NLB) was significantly affected by all included fixed factors, except for period × time of day × farm (Table 5; Figure 1d and Figure 2c,d). The NLB was lower during nighttime than during daytime in every period (winter: t = 11.36, *p* < 0.001; temperate seasons: t = 11.44, *p* < 0.001; summer: t = 15.10, *p* < 0.001). The daytime NLB was significantly higher during the temperate season compared to summer (t = 3.44, *p* = 0.002), whereas the NLB in winter did not significantly differ from the other periods (vs. temperate: t = 2.32, *p* = 0.053; vs. summer: t = −0.20, *p* = 0.979). The nighttime NLB is significantly higher in temperate seasons compared to winter (t = 2.38, *p* = 0.046), whereas the NLB in summer did not significantly differ from the other periods (vs. winter: t = 2.25, *p* = 0.064; vs. temperate: t = 0.21, *p* = 0.976). During both the nighttime and the daytime there were significant differences between farms in NLB (Figure 2c,d; Supplementary Materials, Table S1). In temperate seasons and summer, the NLB was significantly lower on farm E than on most other farms, whereas it was significantly higher on farms C and H than on most other farms. In general, there were less significant differences between farms during winter compared to summer and temperate seasons. The effect of the period on NLB also differed between farms (Supplementary Materials, Table S2). Farms A, C, H and B (nighttime only) showed a significant increase from winter to temperate seasons. On the other hand, farms D, F, G and B (daytime only) showed no significant change between periods. Farm E showed a decrease from winter to spring and summer. Internal THI had no significant effects on NLB (day: t = 0.81, *p* = 0.420; night: t = −0.90, *p* = 0.371; Table 4).

The mean lying bout duration (MLBD) was significantly affected by period, farm, period × farm and time of day × farm (Table 5; Figure 1f and Figure 2e,f). There was no significant difference between daytime and nighttime in the MLBD in any of the periods (winter: t = 1.30, *p* = 0.194; temperate seasons: t = −1.92, *p* = 0.055; summer: t = 0.73, *p* = 0.463). During both daytime and nighttime, the MLBD is significantly lower in temperate seasons and summer compared to winter (day: vs. temperate: t = −5.61, *p* < 0.001; vs. summer: t = −7.91, *p* < 0.001; night: vs. temperate t = −8.27, *p* < 0.001; vs. summer: t = −8.40, *p* < 0.001). The daytime MLBD is also significantly lower during summer compared to temperate seasons (t = 2.63, *p* = 0.023). There were more significant differences in the MLBD between farms during the nighttime compared to the daytime and in the temperate season compared to summer and winter (Figure 2e,f; Supplementary Materials, Table S1). Farm C had (especially during the nighttime) lower MLBD values compared to many other farms. In contrast, farm E had (mainly during the temperate season) higher values compared to many other farms. Most farms showed a decrease in MLBD from winter to temperate and summer seasons (although not always significantly; Supplementary Materials, Table S2). However, farm C (nighttime) and farm D (daytime) had no significant differences between periods and farm E (daytime and nighttime) had no significant differences between winter and temperate seasons. The internal THI had a significant negative effect on both daytime and nighttime MLBD (day: t = −4.43, *p* < 0.001; night: t = −4.44, *p* < 0.477; Table 4).

## 4. Discussion

### 4.1. Effect of Period of the Year and Time of Day on Cow Behavior

In accordance with previous reports (e.g., [26,27]), we found that the behavior varied depending on the time of day. Previously, Veissier and colleagues [55,56,57] found that cattle are more active during the day than during the night. Other findings show that cows lie down more during the night than during the day [58,59]. Our findings are in line with these previous reports. We found that all observed behaviors were shown during both daytime and nighttime. However, cows lie down more during the nighttime, whereas feeding is more concentrated during the daytime. Standing was also more prevalent during the daytime, but only in temperate seasons and summer. Since feed delivery and milking are concentrated during the daytime, this could have affected these patterns. Moreover, it must be noted that the fixed intervals for nighttime and daytime meant that these intervals did not exactly correspond to daylight vs. dark hours, depending on the period. For instance, during summer, there was some daylight at the end of the nighttime. Still, this methodology is in line with previous studies (e.g., [57,59]), resulting in comparable findings. Interestingly, the mean lying bout duration did not significantly differ between daytime and nighttime. This means that the concentration of lying during the nighttime is caused by an increased frequency of lying bouts, indicating shorter intervals between bouts. The period had little effect on these day–night differences, except for standing, which is not significantly diurnal in the winter. Stress has been suggested to disrupt circadian rhythms [56,60]. However, this has not yet been reported for heat stress in cows. Our findings do not allow us to suggest such an effect of heat stress on the circadian rhythm. However, this might only occur during more severe heat stress conditions.

Both daytime and nighttime behavioral patterns were affected by period. During the daytime, the CLI, LD and MLBD are significantly lower in the summer compared to winter. This is in line with previous reports (e.g., [13,61]) and suggests that the hotter weather during summer reduced the lying time. Indeed, internal THI had significant negative effects on CLI, LD and MLBD, which is in line with previous reports (reviewed by [4,62]). This suggests that the differences between the periods are at least in part due to changes in THI. In contrast, the CSI increased from winter to temperate seasons and summer and was positively affected by internal THI. This is in line with previous reports [16,63] and supports the suggestion that during hot weather, cows rest more while standing to improve evaporative cooling [15]. The effect of period on daytime CFI is more subtle and shows only a significant increase from winter to the temperate season. This contrasts with reports of reduced feed intake during periods of heat stress (e.g., [32,33,64]). However, reductions in feed intake due to heat stress can become less over time due to acclimatization [65]. Since data were collected in the middle of summer, it may be that the cows were sufficiently acclimatized to not show a decrease in feed intake. However, since there is no recording of feed intake during early summer, this is only speculation. The effect on NLB is also not so clear. This suggests that a decrease in lying time due to heat stress was primarily due to decreases in mean lying bout duration, as also reported in previous reports [16,63]. A reason for this may be that heat loss from the cow’s body to the bedding gradually decreases with time during a lying bout [66], possibly causing the cows to rise earlier during heat stress conditions. Previously, it was reported that nighttime behavioral patterns are not affected by hot weather [11,26,29]. We found, however, that apart from CLI, all behavioral parameters differed significantly between the periods during the nighttime. This shows that nighttime behavior can also be affected by period of the year. This difference compared to previous studies might be partially explained by the relatively high THI levels that were recorded during the summer nights (73.8 ± 2.4). These values were above the heat stress threshold of 72 [34] and above a threshold of 67, which is the threshold for cows to reduce lying time [13]. Indeed, THI had a significant negative effect on the LD and MLBD at night. This suggests that the differences between periods in these parameters are at least in part due to changes in THI. However, THI had no significant effect on nighttime CSI, CFI and NLB, even though these changed significantly between periods. This means that factors other than THI induced these changes. Farmed animal behavior is known to change between seasons, and these changes are ascribed to a combination of environmental (e.g., temperature and photoperiod) and endogenous (e.g., annuality) factors [37]. Since in this study the temperate season included both measurements in spring and autumn, further studies focusing on all four seasons are needed to better understand the effect of seasons on cow behavior. Nighttime differences between periods in CSI, LD and MLBD are comparable to the differences between periods during the day. Differences between periods in nighttime CFI and NLB were more subtle. CFI only decreased in summer compared to winter and temperate seasons, and NLB increased during temperate seasons compared to winter. Altogether, these findings show that nighttime behavior can also be affected by period and THI. Thus, during warmer periods, cows do not seem able to recover during the night from the loss in lying time they build up during the day. These results emphasize the need to monitor both night and daytime behavior to better understand how hot weather conditions may affect cow behavior.

Even though both CLI and LD aim to estimate the total lying time of all cows in a farm, the two measures yielded somewhat different results. Both parameters are commonly used as measures of cow lying behavior (CLI: [26,51,67]; LD: [53,54,68]). The accuracy of these measures depends on the frequency of measurement and the number of animals included. In this study, picture data were analyzed every hour for the entire group, whereas accelerometer data were analyzed every minute for only a sample of the group (ten subjects per farm). In this case, it is, therefore, difficult to determine which of these parameters better reflects the actual total lying time of all cows.

### 4.2. Effect of Housing on Cow Behavior

Housing had a significant effect on all three parameters measured with the use of accelerometers, as well as significant interactions with period. The interactions of housing with both period and time of day were only significant for LD. Although not reported here, the total daily time averaged 12.3 (±1.2) in winter, 10.5 (±1.4) in temperate seasons and 9.7 (±1.3) in summer (see Supplementary Materials, Table S3 and [48] for further details). In summer, farms A, B, E, F, G and H showed values below 10 h/d, constituting potential welfare risks [4]. This suggests that these barn structures are insufficient in protecting the cows from the hot weather conditions that occur during summers in Northern Italy. Previous publications on the same farms reported that several of these farms also showed much higher internal THI values compared to external THI values [48,49]. This could be caused by structural problems of the barn, such as insufficient roof insulation [48,49]. Considering that due to global warming, heat stress is becoming an increasingly more important welfare issue [62], urgent efforts need to be made to better understand how the barn structure affects cow lying behavior and welfare and to provide this information to dairy cow farmers. For LD, the main variation between farms was found during the daytime, whereas nighttime values did not vary much between farms. This means that the daytime variation between farms in LD is mainly affected by factors that happen only during the day. These factors can be climatic (as will be discussed further on) as well as caused by farm management. Structural aspects that do not greatly affect the barn climate, such as pen size and stocking density, are, therefore, less likely to account for these differences since they would affect LD during both daytime and nighttime. It must also be noted that the number of cows per cubicle in all farms was around or below 1.0, whereas most studies only report effects when a value of 1.2 is exceeded [4]. Therefore, overstocking does not seem to be a major issue here. Additionally, since we found that THI significantly affected nighttime LD, this cannot be due to nighttime lying being less dependent on climate conditions, as previously reported [11,26]. Alternatively, it may be that differences in daytime management cause these effects. Previous studies have reported that the frequency of feed delivery and the length of milking may be negatively correlated to lying time [45,47]. In this study, the farms with the lowest LD values (B and E) had only one feed delivery per day and 30 min for milking, whereas the farms with the highest LD values had both longer milking durations (D: 35 min and F: 45 min) and one of these farms (D) had two feed deliveries per day (see Table 2). Therefore, these factors do not seem to explain the observed differences in LD. Alternatively, the timing of feed delivery, the frequency of pushing up feed and the presence of staff and their interactions with the cows can also affect cow behavior and welfare (e.g., [42,69,70]) and, therefore, could have caused farm differences in LD during the daytime. Further research is needed to test this. In contrast to other farms, farm D showed no significant change in daytime LD between the periods. In a previous report on this project [48], this farm was identified as one of four farms with lower internal THI values in summer. As reported by Lovarelli et al. [49], certain barn structural elements have a significant effect on the internal THI level. They found that the presence of roof insulation, forced ventilation in the feeding and resting area and an E–W/NE–SW orientation resulted in significantly lower internal THI levels. In contrast, a roof height below 7 m and only partial (vs. complete) lateral openings resulted in significantly higher internal THI levels [49]. Farm D had roof insulation, a NE–SW orientation, a high roof height (7.7 m) and forced ventilation in the feeding area, which could account for the low THI levels in summer. However, it had only partial lateral openings and no forced ventilation in the resting area. Furthermore, other farms (e.g., farm E) also had many of the above-mentioned structural elements and, despite presenting relatively low internal THI values in summer, they did not show similar stability in lying time. For this reason, it seems that barn THI values alone cannot account for these different LD patterns. In addition to THI, researchers have also suggested that windspeed contributes to the heat stress experienced by the animal [71,72]. Wind speed affects the rate of heat loss, resulting in lower respiration rates [71,73]. Therefore, structural elements that improve the air flow through the barn, such as ridge openings, roof slope and well-positioned lateral openings [74,75], could help cows to lose heat during warm weather conditions. This could reduce the need for cows to stand while resting (instead of lying down) to improve evaporative cooling [15]. Considering that the lying behavior of cows can be affected by many environmental and animal-based factors [4], lying could be considered more a general indicator of cow welfare rather than a specific indicator of heat stress [5]. In future research, it is, therefore, important to improve our understanding of all factors that may affect cow lying time and to account for them when measuring lying time as an indicator of cow welfare.

In contrast to LD, the significant farm differences in NLB and MLBD were apparent during both daytime and nighttime. In both sets of data, a contrast can be discerned between some farms with more lying bouts of shorter duration (primarily farm C, but to some extent also farm H) and other farms with fewer lying bouts of longer duration (primarily farm E). It has been reported in previous work that uncomfortable lying surfaces (e.g., hard, wet or dirty) result in fewer lying bouts and longer lying bout duration [44,76]. Both farm C (most lying bouts) and farm E (fewest lying bouts) had similar cubicle dimensions (1.8 × 1.2 m) and used mattresses as cubicle bedding. Therefore, cubicle dimensions and the type of bedding material may not be the cause. However, other factors such as the condition of the mattresses (e.g., cleanliness and wear) and cow health may also play a role. Indeed, health checks were performed on each observed group at the start and the end of each data collection period. On farm E, a few cases of lameness were noted, as well as many cows that were dirty. In contrast, on farm C, no cases of lameness were found, and only a few animals were found to be dirty. Since both lameness and dirty lying surfaces have been found to result in fewer but longer lying bouts [44,77], this may account for the current findings. Farms also differed in the way NLB and MLBD were affected by period. The patterns for MLBD were similar to those for LD, with most farms showing a decrease from winter to temperate seasons and summer. For NLB, the patterns differed between farms. Some farms (mainly farms A, C and H) showed an increase from winter to temperate seasons and/or summer, whereas farm E showed a decrease and farms D, F and G showed no significant change. Farms A, C and H (as well as B) were identified by Lovarelli et al. [48,49] to have high internal THI levels, whereas the other farms had lower levels. Therefore, it could be that this increase in NLB is related to these higher THI levels and may be a way to compensate for the decrease in MLBD in these farms. However, since NLB is not found to be significantly affected by THI in this study, as well as previous reports [16,63], this explanation seems unlikely. Further research is, therefore, required to better understand which factors could cause this different response between farms.

Lying, feeding and standing are all behaviors that have been found to play a central role in cow welfare assessment (for reviews see, e.g., [4,5,78]), since they can be both the cause and consequence of poor welfare conditions (e.g., heat stress and lameness). The findings reported here provide further support for this notion, especially regarding lying behavior. An advantage of these studied behaviors (as opposed to more complex behavior, such as most social interactions) is that they can also be relatively easily monitored using automatic methods, such as accelerometers. Indeed, many commercial systems have already been developed to measure these behaviors (see [79] for a review). This means that farmers would be able to use this information on the farm in their real-time assessment of a cow’s wellbeing. Combining such behavioral measurements with climate monitoring, as is advised under the concept of precision livestock farming, would further improve the on-farm assessment of cow welfare.

## 5. Conclusions

The results of this study show that cow behavior is affected by the period of the year, the time of day and housing. Although the effects of the period and time of day on some behavioral parameters may be partially ascribed to differences in THI, our results show that THI alone cannot account for these effects in cow behavior and that other environmental (e.g., photoperiod) and endogenous (e.g., circadian rhythms) factors may also play an important role. In contrast to previous reports, we also found that nighttime behavior is affected by both the period of the year and housing. Therefore, this period of the day should not be ignored when studying cow behavior as an indicator of cow welfare. In addition, our findings also show significant variation between farms in all measured behavioral parameters. These farm differences may be linked to differences in barn structure and farm management, showing the importance of further research to better understand which measures farmers can take to improve the housing conditions for dairy cows. These findings also stress the importance of monitoring cow behavior, in addition to climate monitoring, to assess cow welfare on the farm more accurately.

## Figures and Tables

**Table 1:** Selected farm properties. THI (temperature-humidity index) values are means per farm as measured during the three sampling periods.

Variable	Farm A	Farm B	Farm C	Farm D	Farm E	Farm F	Farm G	Farm H
Mean internal THI winter	42.54	42.61	44.16	45.01	44.18	46.48	49.38	48.27
Mean internal THI temperate	66.04	66.24	63.66	63.66	66.04	66.32	62.07	61.84
Mean internal THI summer	78.49	78.67	74.41	73.63	73.85	74.41	75.25	75.31
Herd size (N)	350	800	830	900	200	630	270	475
Duration of milking (min./day)	45	30	35	35	30	45	45	30
Feeding times (No./day)	2	1	2	2	1	1	1	1
Barn orientation	E-W	NW-SE	NE-SW	E-W	NW-SE	NW-SE	NW-SE	E-W
Ridge height (m)	7.0	12.2	7.7	6.5	7.0	13.4	5.4	7.5
Roof insulation	Yes	No	No	Yes	Yes	Yes	Yes	Yes
Cooling in feeding area	Yes	Yes	No	Yes	Yes	No	Yes	Yes
Cooling in resting area	Yes	No	Yes	No	Yes	Yes	Yes	Yes
Monitored surface area (m^2^)	492	3472	586	3825	890	2464	939	843
Monitored group size (N)	61	113	70	144	35	144	54	88
Stocking density (m^2^/cow)	8.07	30.73	8.37	26.56	25.43	17.11	17.39	9.58
No. of cows per cubicle	0.92	0.91	1.01	0.99	0.83	0.92	1.04	0.8
Cubicle length: body (m)	1.8	1.8	1.8	1.85	1.8	1.75	1.95	1.7
Cubicle width (m)	1.25	1.2	1.2	1.25	1.2	1.2	1.2	1.2
Bedding material	straw	straw	mattress	mattress	mattress	straw	sand	mattress
Floor type	slatted-grooved	slatted	slatted	grooved	rubber	grooved	grooved	grooved

**Table 2:** Description and calculation of the parameters that were included in this study. The parameters measured with the use of accelerometers were calculated for two periods per day, i.e., daytime and nighttime, separately. All other indexes were calculated per hour and averaged for daytime and nighttime. In the THI formula, T indicates ambient temperature (°C) and RH indicates relative humidity (%).

Type of Data	Parameter	Abbreviation	Description/Calculation
Climate	Temperature-humidity index	THI	THI = 0.81 × T + 0.143 × RH + 0.0099 × RH × T + 46.3
Picture	Cow lying index	CLI	No. of cows lying down/total no. of cows
Picture	Cow standing index	CSI	No. of cows standing/total no. of cows
Picture	Cow feeding index	CFI	No. of cows feeding/total no. of cows
Accelerometer	Lying duration (%)	LD	Hours spent lying/total hours × 100
Accelerometer	Number of lying bouts (*n*/h)	NLB	Number of lying bouts initiated/total hours
Accelerometer	Mean lying bout duration (min)	MLBD	Minutes spent lying/Number of lying bouts

**Table 3:** F and *p*-values for each fixed effect that was included in the GLIMMIX model testing CLI (cow lying index), CSI (cow standing index) and CFI (cow feeding index). The significance level is at < 0.05.

Fixed Effect	CLI		CSI		CFI	
	F	*p*	F	*p*	F	*p*
Period	20.46	<0.001	42.36	<0.001	7.39	0.001
Time of day	184.20	<0.001	32.51	<0.001	289.55	<0.001
Period × Time of day	15.00	<0.001	10.17	<0.001	7.80	0.001

**Table 4:** Estimates, standard errors, degrees of freedom (DF), t and *p*-values for the effect of THI on the CLI (cow lying index), CSI (cow standing index), CFI (cow feeding index), LD (lying duration), NLB (number of lying bouts) and MLBD (mean lying bout duration). The significance level is at < 0.05.

Time of Day	Parameter	Estimate	Std. Err.	DF	t	*p*
Nighttime	CLI	−0.0007	0.0009	22.35	−0.72	0.477
	CSI	0.0014	0.0007	19.25	2.06	0.053
	CFI	−0.0010	0.0005	17.05	−1.97	0.065
	LD	−0.2271	0.0510	262.2	−4.46	<0.001
	NLB	0.0006	0.0008	296.2	0.81	0.420
	MLBD	−0.5497	0.1242	214.8	−4.43	<0.001
Daytime	CLI	−0.0044	0.0011	20.7	−4.16	<0.001
	CSI	0.0047	0.0008	17.93	5.75	<0.001
	CFI	−0.0003	0.0005	16.21	−0.57	0.579
	LD	−0.3321	0.0486	324.5	−6.84	<0.001
	NLB	−0.0006	0.0006	298.8	−0.90	0.371
	MLBD	−0.5323	0.1199	245.6	−4.44	<0.001

**Table 5:** F and *p*-values for each fixed effect that was included in the GLIMMIX model testing LD (lying duration), NLB (number of lying bouts) and MLBD (mean lying bout duration). The significance level is at < 0.05.

Fixed Effect	LD		NLB		MLBD	
	F	*p*	F	*p*	F	*p*
Period	115.67	<0.001	5.18	0.006	52.53	<0.001
Time of day	673.48	<0.001	477.29	< 0.001	0.00	0.955
Farm	6.48	<0.001	20.32	< 0.001	12.32	<0.001
Period × Time of day	21.00	<0.001	3.84	0.022	2.95	0.053
Period × Farm	15.49	<0.001	6.97	< 0.001	4.32	<0.001
Time of day × Farm	25.71	<0.001	11.83	< 0.001	6.64	<0.001
Period × Time of day × Farm	4.32	<0.001	1.62	0.067	0.79	0.681

## Data Availability

The raw data supporting the conclusions of this article will be made available by the authors, without undue reservation.

## Supplementary Materials

The following supporting information can be downloaded at: https://www.mdpi.com/article/10.3390/ani12040512/s1, Table S1: t and *p*-values of the pairwise comparisons of farms within each period and time of day; Table S2: t and *p*-values of the pairwise comparisons of periods for each farm; Table S3: Means of daily values per farm and per period for lying duration (LD), number of lying bouts (NLB) and mean lying bout duration (MLBD).
