# Supplementary material for: Dairy Cow Behavior Is Affected by Period, Time of Day and Housing
Source: Animals (Basel). 2022 Feb 18;12(4):512. doi: 10.3390/ani12040512 (PMC8868199; doi:10.3390/ani12040512)
Supplement: Supplementary file 1 [file animals-12-00512-s001.zip › Leliveld_TableS1.pdf]

Table S1: t- and *p*-values of the pairwise comparisons of farms within each period and time of day (Tukey-Kramer test, slice-option). Significant differences ($p < 0.05$) are highlighted in bold.

**Period**	**Time of day**	**Farm1**	**Farm2**	**LD**		**NLB**		**MLBD**	
				**t**	***p***	**t**	***p***	**t**	***p***
Winter	Nighttime	F	G	2.03	0.461	0.83	0.991	-1.87	0.568
Winter	Nighttime	F	H	-1.21	0.928	-0.88	0.988	-2.46	0.212
Winter	Nighttime	F	C	2.35	0.269	-2.54	0.181	2.74	0.111
Winter	Nighttime	F	B	-0.16	1.000	2.35	0.269	**-3.45**	**0.013**
Winter	Nighttime	F	E	1.55	0.782	-1.50	0.805	**-3.35**	**0.019**
Winter	Nighttime	F	A	0.87	0.989	1.04	0.968	-0.53	1.000
Winter	Nighttime	F	D	1.29	0.901	**3.17**	**0.033**	-1.01	0.974
Winter	Nighttime	G	H	-2.85	0.083	-1.49	0.814	-0.66	0.998
Winter	Nighttime	G	C	0.62	0.999	-2.98	0.058	**4.28**	**0.001**
Winter	Nighttime	G	B	-1.70	0.687	1.64	0.728	-1.71	0.681
Winter	Nighttime	G	E	-0.42	1.000	-2.08	0.428	-1.42	0.849
Winter	Nighttime	G	A	-0.93	0.983	0.27	1.000	1.21	0.931
Winter	Nighttime	G	D	-0.55	0.999	2.16	0.379	0.73	0.996
Winter	Nighttime	H	C	**3.37**	**0.017**	-1.68	0.703	**4.97**	**<0.001**
Winter	Nighttime	H	B	0.89	0.987	3.03	0.051	-1.09	0.958
Winter	Nighttime	H	E	**3.09**	**0.043**	-0.58	0.999	-0.79	0.994
Winter	Nighttime	H	A	2.06	0.444	1.90	0.551	1.89	0.558
Winter	Nighttime	H	D	2.42	0.230	**3.87**	**0.003**	1.40	0.857
Winter	Nighttime	C	B	-2.22	0.342	**4.30**	**<0.001**	**-5.78**	**<0.001**
Winter	Nighttime	C	E	-1.08	0.961	1.36	0.876	**-5.86**	**<0.001**
Winter	Nighttime	C	A	-1.54	0.786	**3.42**	**0.015**	**-3.17**	**0.033**
Winter	Nighttime	C	D	-1.31	0.896	**6.01**	**<0.001**	**-3.81**	**0.004**
Winter	Nighttime	B	E	1.46	0.827	**-3.62**	**0.007**	0.43	1.000
Winter	Nighttime	B	A	0.91	0.985	-1.48	0.819	2.88	0.076
Winter	Nighttime	B	D	1.25	0.917	0.20	1.000	2.41	0.238
Winter	Nighttime	E	A	-0.62	0.999	2.67	0.134	2.74	0.111
Winter	Nighttime	E	D	-0.18	1.000	**4.91**	**<0.001**	2.22	0.339
Winter	Nighttime	A	D	0.40	1.000	2.03	0.465	-0.47	1.000
Winter	Daytime	F	G	2.51	0.189	-0.04	1.000	-0.58	0.999
Winter	Daytime	F	H	0.40	1.000	-2.09	0.421	0.41	1.000
Winter	Daytime	F	C	-0.19	1.000	-1.52	0.796	1.78	0.637
Winter	Daytime	F	B	1.74	0.658	1.69	0.692	-2.31	0.290
Winter	Daytime	F	E	**7.30**	**<0.001**	0.07	1.000	-0.90	0.986
Winter	Daytime	F	A	**4.34**	**<0.001**	-0.01	1.000	2.19	0.355
Winter	Daytime	F	D	1.52	0.795	2.80	0.094	-0.21	1.000
Winter	Daytime	G	H	-1.75	0.657	-1.89	0.555	0.93	0.983
Winter	Daytime	G	C	-2.16	0.378	-1.38	0.864	2.22	0.340
Winter	Daytime	G	B	-0.25	1.000	1.64	0.729	-1.73	0.665
Winter	Daytime	G	E	**4.42**	**<0.001**	0.10	1.000	-0.31	1.000
Winter	Daytime	G	A	1.89	0.559	0.03	1.000	2.64	0.141
Winter	Daytime	G	D	-0.75	0.995	2.56	0.173	0.32	1.000
Winter	Daytime	H	C	-0.55	0.999	0.37	1.000	1.36	0.877
Winter	Daytime	H	B	1.36	0.876	**3.39**	**0.016**	-2.64	0.141
Winter	Daytime	H	E	**7.38**	**<0.001**	2.64	0.142	-1.37	0.869
Winter	Daytime	H	A	**3.83**	**0.003**	2.08	0.427	1.74	0.658
Winter	Daytime	H	D	1.05	0.966	**4.72**	**<0.001**	-0.60	0.999
Winter	Daytime	C	B	1.77	0.639	2.84	0.086	**-3.81**	**0.003**

Winter	Daytime	C	E	**6.60**	**<0.001**	1.65	0.721	-2.64	0.143
Winter	Daytime	C	A	**4.07**	**0.001**	1.49	0.813	0.30	1.000
Winter	Daytime	C	D	1.71	0.679	**4.50**	**<0.001**	-2.03	0.461
Winter	Daytime	B	E	**4.31**	**<0.001**	-1.70	0.688	1.52	0.799
Winter	Daytime	B	A	2.01	0.477	-1.70	0.689	**4.29**	**<0.001**
Winter	Daytime	B	D	-0.43	1.000	0.56	0.999	2.04	0.455
Winter	Daytime	E	A	-2.66	0.134	-0.08	1.000	3.15	0.035
Winter	Daytime	E	D	**-5.67**	**<0.001**	2.93	0.067	0.67	0.998
Winter	Daytime	A	D	-2.78	0.101	2.72	0.117	-2.32	0.284
Temperate	Nighttime	F	G	**-4.39**	**<0.001**	0.28	1.000	-2.88	0.076
Temperate	Nighttime	F	H	**-6.15**	**<0.001**	**-3.16**	**0.034**	-2.02	0.470
Temperate	Nighttime	F	C	-0.34	1.000	**-6.76**	**<0.001**	2.31	0.290
Temperate	Nighttime	F	B	0.05	1.000	0.88	0.988	-1.43	0.845
Temperate	Nighttime	F	E	-2.60	0.158	**4.02**	**0.002**	**-5.51**	**<0.001**
Temperate	Nighttime	F	A	**-3.40**	**0.015**	-1.52	0.798	-0.87	0.989
Temperate	Nighttime	F	D	**-3.21**	**0.029**	1.02	0.972	-1.44	0.837
Temperate	Nighttime	G	H	-1.78	0.636	**-3.20**	**0.031**	0.85	0.990
Temperate	Nighttime	G	C	**3.54**	**0.010**	**-6.63**	**<0.001**	**5.01**	**<0.001**
Temperate	Nighttime	G	B	**3.63**	**0.007**	0.64	0.998	1.26	0.913
Temperate	Nighttime	G	E	1.72	0.671	**3.42**	**0.014**	-2.54	0.179
Temperate	Nighttime	G	A	0.56	0.999	-1.67	0.707	1.75	0.651
Temperate	Nighttime	G	D	1.51	0.800	0.60	0.999	1.57	0.771
Temperate	Nighttime	H	C	**5.58**	**<0.001**	**-3.90**	**0.003**	**4.33**	**<0.001**
Temperate	Nighttime	H	B	**5.48**	**<0.001**	**3.55**	**0.009**	0.48	1.000
Temperate	Nighttime	H	E	**3.97**	**0.002**	**7.80**	**<0.001**	**-3.57**	**0.009**
Temperate	Nighttime	H	A	2.38	0.249	1.46	0.827	1.00	0.974
Temperate	Nighttime	H	D	**3.72**	**0.005**	**4.55**	**<0.001**	0.72	0.996
Temperate	Nighttime	C	B	0.36	1.000	**6.69**	**<0.001**	**-3.62**	**0.007**
Temperate	Nighttime	C	E	-2.16	0.380	**11.26**	**<0.001**	**-7.80**	**<0.001**
Temperate	Nighttime	C	A	**-3.51**	**0.011**	**6.27**	**<0.001**	**-3.26**	**0.025**
Temperate	Nighttime	C	D	-2.46	0.214	**8.22**	**<0.001**	**-3.76**	**0.004**
Temperate	Nighttime	B	E	-2.29	0.298	2.35	0.267	**-3.77**	**0.004**
Temperate	Nighttime	B	A	**-3.11**	**0.040**	-2.16	0.374	0.51	1.000
Temperate	Nighttime	B	D	-2.61	0.153	-0.18	1.000	0.18	1.000
Temperate	Nighttime	E	A	-1.17	0.942	**-5.66**	**<0.001**	**4.31**	**<0.001**
Temperate	Nighttime	E	D	-0.35	1.000	**-3.84**	**0.003**	**4.60**	**<0.001**
Temperate	Nighttime	A	D	0.87	0.989	2.54	0.181	-0.38	1.000
Temperate	Daytime	F	G	-1.25	0.917	0.61	0.999	-2.20	0.354
Temperate	Daytime	F	H	**-3.49**	**0.012**	**-4.06**	**0.001**	0.55	0.999
Temperate	Daytime	F	C	-0.09	1.000	**-3.36**	**0.018**	2.40	0.241
Temperate	Daytime	F	B	-0.01	1.000	0.95	0.981	-1.23	0.922
Temperate	Daytime	F	E	1.88	0.564	**4.38**	**<0.001**	**-4.78**	**<0.001**
Temperate	Daytime	F	A	1.19	0.935	-1.54	0.785	1.93	0.531
Temperate	Daytime	F	D	-1.45	0.836	2.57	0.166	-1.10	0.956
Temperate	Daytime	G	H	-2.16	0.378	**-4.34**	**<0.001**	2.69	0.125
Temperate	Daytime	G	C	1.02	0.972	**-3.70**	**0.005**	**4.45**	**<0.001**
Temperate	Daytime	G	B	1.00	0.974	0.45	1.000	0.82	0.992
Temperate	Daytime	G	E	2.94	0.065	**3.45**	**0.013**	-2.50	0.196
Temperate	Daytime	G	A	2.23	0.337	-1.97	0.503	**3.89**	**0.003**
Temperate	Daytime	G	D	-0.04	1.000	1.62	0.741	1.19	0.935
Temperate	Daytime	H	C	**3.28**	**0.024**	0.44	1.000	1.90	0.552
Temperate	Daytime	H	B	**3.06**	**0.046**	**4.37**	**<0.001**	-1.78	0.634

Temperate	Daytime	H	E	**5.78**	**<0.001**	**9.19**	**<0.001**	**-5.48**	**<0.001**
Temperate	Daytime	H	A	**4.54**	**<0.001**	2.31	0.289	1.44	0.837
Temperate	Daytime	H	D	2.47	0.207	**7.06**	**<0.001**	-1.66	0.712
Temperate	Daytime	C	B	0.06	1.000	**3.82**	**0.003**	**-3.52**	**0.010**
Temperate	Daytime	C	E	1.92	0.536	**7.90**	**<0.001**	-7.17	<0.001
Temperate	Daytime	C	A	1.46	0.829	2.18	0.364	-0.37	1.000
Temperate	Daytime	C	D	-1.18	0.938	**5.85**	**<0.001**	**-3.54**	**0.010**
Temperate	Daytime	B	E	1.64	0.728	2.58	0.165	**-3.27**	**0.024**
Temperate	Daytime	B	A	1.08	0.961	-2.24	0.325	3.02	0.052
Temperate	Daytime	B	D	-1.13	0.950	0.89	0.987	0.29	1.000
Temperate	Daytime	E	A	-0.55	0.999	**-6.04**	**<0.001**	**6.51**	**<0.001**
Temperate	Daytime	E	D	**-3.79**	**0.004**	-2.58	0.165	**4.14**	**0.001**
Temperate	Daytime	A	D	-2.54	0.179	**3.89**	**0.003**	-2.99	0.057
Summer	Nighttime	F	G	1.20	0.933	0.35	1.000	-0.74	0.996
Summer	Nighttime	F	H	0.45	1.000	-2.85	0.084	-0.46	1.000
Summer	Nighttime	F	C	-0.22	1.000	**-5.69**	**<0.001**	1.99	0.486
Summer	Nighttime	F	B	1.28	0.907	0.95	0.981	-1.42	0.847
Summer	Nighttime	F	E	0.59	0.999	2.73	0.114	-2.99	0.056
Summer	Nighttime	F	A	-0.05	1.000	0.19	1.000	-0.83	0.991
Summer	Nighttime	F	D	-1.75	0.654	1.73	0.669	-1.53	0.791
Summer	Nighttime	G	H	-0.66	0.998	-2.98	0.059	0.26	1.000
Summer	Nighttime	G	C	-1.27	0.911	**-5.68**	**<0.001**	2.67	0.133
Summer	Nighttime	G	B	0.26	1.000	0.64	0.998	-0.71	0.997
Summer	Nighttime	G	E	-0.59	0.999	2.17	0.370	-2.18	0.363
Summer	Nighttime	G	A	-1.14	0.949	-0.13	1.000	-0.10	1.000
Summer	Nighttime	G	D	-2.75	0.110	1.19	0.934	-0.72	0.996
Summer	Nighttime	H	C	-0.66	0.998	-3.03	0.050	2.45	0.219
Summer	Nighttime	H	B	0.89	0.987	**3.35**	**0.018**	-0.98	0.978
Summer	Nighttime	H	E	0.11	1.000	**6.11**	**<0.001**	-2.54	0.178
Summer	Nighttime	H	A	-0.50	1.000	**3.04**	**0.049**	-0.37	1.000
Summer	Nighttime	H	D	-2.21	0.348	**4.86**	**<0.001**	-1.02	0.972
Summer	Nighttime	C	B	1.45	0.835	**5.83**	**<0.001**	**-3.31**	**0.021**
Summer	Nighttime	C	E	0.81	0.993	**8.89**	**<0.001**	**-5.01**	**<0.001**
Summer	Nighttime	C	A	0.20	1.000	**7.41**	**<0.001**	**-3.06**	**0.047**
Summer	Nighttime	C	D	-1.36	0.874	**7.64**	**<0.001**	**-3.52**	**0.010**
Summer	Nighttime	B	E	-0.82	0.992	1.23	0.924	-1.38	0.866
Summer	Nighttime	B	A	-1.33	0.888	-0.79	0.994	0.63	0.998
Summer	Nighttime	B	D	-2.78	0.101	0.34	1.000	0.04	1.000
Summer	Nighttime	E	A	-0.67	0.998	-2.62	0.148	2.18	0.365
Summer	Nighttime	E	D	-2.66	0.134	-1.34	0.884	1.64	0.723
Summer	Nighttime	A	D	-1.62	0.741	1.42	0.848	-0.64	0.998
Summer	Daytime	F	G	1.68	0.700	0.21	1.000	-0.80	0.993
Summer	Daytime	F	H	**3.06**	**0.046**	-1.83	0.601	1.45	0.835
Summer	Daytime	F	C	0.77	0.994	-1.98	0.494	1.77	0.637
Summer	Daytime	F	B	**4.36**	**<0.001**	1.75	0.652	0.77	0.994
Summer	Daytime	F	E	**5.39**	**<0.001**	**4.22**	**0.001**	-2.03	0.459
Summer	Daytime	F	A	2.37	0.257	-0.02	1.000	1.32	0.890
Summer	Daytime	F	D	-0.93	0.983	2.53	0.183	-1.22	0.925
Summer	Daytime	G	H	1.38	0.867	-1.90	0.552	2.18	0.361
Summer	Daytime	G	C	-0.74	0.996	-2.06	0.443	2.50	0.196
Summer	Daytime	G	B	2.85	0.084	1.52	0.796	1.49	0.810
Summer	Daytime	G	E	**3.44**	**0.014**	**3.66**	**0.006**	-1.20	0.934

Summer	Daytime	G	A	0.73	0.996	-0.21	1.000	2.07	0.434
Summer	Daytime	G	D	-2.47	0.210	2.03	0.460	-0.38	1.000
Summer	Daytime	H	C	-2.18	0.365	-0.26	1.000	0.35	1.000
Summer	Daytime	H	B	1.68	0.700	**3.30**	**0.022**	-0.61	0.999
Summer	Daytime	H	E	2.22	0.338	**6.52**	**<0.001**	**-3.57**	**0.009**
Summer	Daytime	H	A	-0.68	0.998	1.80	0.618	-0.13	1.000
Summer	Daytime	H	D	**-4.25**	**0.001**	**4.51**	**<0.001**	-2.71	0.120
Summer	Daytime	C	B	**3.57**	**0.009**	**3.42**	**0.015**	-0.94	0.982
Summer	Daytime	C	E	**4.40**	**<0.001**	**6.25**	**<0.001**	**-3.84**	**0.003**
Summer	Daytime	C	A	1.81	0.613	2.47	0.207	-0.52	1.000
Summer	Daytime	C	D	-1.67	0.708	**4.38**	**<0.001**	-3.00	0.055
Summer	Daytime	B	E	0.10	1.000	1.59	0.755	-2.71	0.119
Summer	Daytime	B	A	-2.27	0.309	-1.78	0.637	0.49	1.000
Summer	Daytime	B	D	**-5.32**	**<0.001**	0.11	1.000	-1.93	0.531
Summer	Daytime	E	A	-2.95	0.063	**-4.41**	**<0.001**	**3.46**	**0.013**
Summer	Daytime	E	D	**-7.40**	**<0.001**	-2.23	0.332	0.92	0.984
Summer	Daytime	A	D	**-3.42**	**0.015**	2.40	0.240	-2.57	0.167
