# Supplementary material for: Dairy Cow Behavior Is Affected by Period, Time of Day and Housing
Source: Animals (Basel). 2022 Feb 18;12(4):512. doi: 10.3390/ani12040512 (PMC8868199; doi:10.3390/ani12040512)
Supplement: Supplementary file 1 [file animals-12-00512-s001.zip › Leliveld_TableS2.pdf]

Table S2: t- and *p*-values of the pairwise comparisons of periods for each farm (Tukey-Kramer test, slice-option). Significant differences ($p < 0.05$) are highlighted in bold.

| Farm | Time of day | Period1 | Period2 | DLT | | NLB | | MLBD | |
|---|---|---|---|---|---|---|---|---|---|
| | | | | t | *p* | t | *p* | t | *p* |
| F | Night | Temperate | Summer | **-4.70** | **<0.001** | -0.56 | 0.839 | -1.02 | 0.564 |
| F | Night | Temperate | Winter | **-10.87** | **<0.001** | -0.67 | 0.778 | **-3.70** | **0.001** |
| F | Night | Summer | Winter | **-6.58** | **<0.001** | -0.16 | 0.986 | **-2.76** | **0.016** |
| G | Night | Temperate | Summer | **4.41** | **<0.001** | -0.43 | 0.905 | 1.97 | 0.120 |
| G | Night | Temperate | Winter | -1.49 | 0.296 | 0.12 | 0.992 | **-2.37** | **0.047** |
| G | Night | Summer | Winter | **-5.93** | **<0.001** | 0.55 | 0.846 | **-4.38** | **<0.001** |
| H | Night | Temperate | Summer | **5.75** | **<0.001** | -0.06 | 0.998 | 1.05 | 0.544 |
| H | Night | Temperate | Winter | **-2.77** | **0.015** | **2.55** | **0.029** | **-3.79** | **<0.001** |
| H | Night | Summer | Winter | **-7.38** | **<0.001** | **2.48** | **0.036** | **-4.54** | **<0.001** |
| C | Night | Temperate | Summer | **-4.37** | **<0.001** | 1.48 | 0.300 | -1.45 | 0.317 |
| C | Night | Temperate | Winter | **-3.57** | **0.001** | **3.39** | **0.002** | -2.11 | 0.088 |
| C | Night | Summer | Winter | -1.27 | 0.415 | **2.68** | **0.020** | -1.15 | 0.482 |
| B | Night | Temperate | Summer | **-2.31** | **0.054** | -0.42 | 0.906 | -1.01 | 0.570 |
| B | Night | Temperate | Winter | **-11.37** | **<0.001** | 2.25 | 0.064 | **-6.52** | **<0.001** |
| B | Night | Summer | Winter | **-9.24** | **<0.001** | **2.64** | **0.023** | **-5.58** | **<0.001** |
| E | Night | Temperate | Summer | 0.46 | 0.888 | **-2.77** | **0.016** | **2.49** | **0.034** |
| E | Night | Temperate | Winter | **-3.93** | **<0.001** | **-7.43** | **<0.001** | -0.64 | 0.799 |
| E | Night | Summer | Winter | **-4.41** | **<0.001** | **-5.55** | **<0.001** | **-2.73** | **0.018** |
| A | Night | Temperate | Summer | 1.17 | 0.468 | 2.34 | 0.050 | -0.76 | 0.730 |
| A | Night | Temperate | Winter | **-3.16** | **0.005** | **3.00** | **0.008** | **-2.59** | **0.026** |
| A | Night | Summer | Winter | **-4.59** | **<0.001** | 1.09 | 0.521 | -2.12 | 0.086 |
| D | Night | Temperate | Summer | **-2.57** | **0.028** | 0.64 | 0.798 | -1.16 | 0.475 |
| D | Night | Temperate | Winter | **-3.69** | **0.001** | 2.33 | 0.052 | **-2.78** | **0.015** |
| D | Night | Summer | Winter | -1.71 | 0.201 | 1.83 | 0.158 | -1.81 | 0.166 |
| F | Day | Temperate | Summer | -1.57 | 0.259 | -0.04 | 0.999 | -0.02 | 1.000 |
| F | Day | Temperate | Winter | **-7.04** | **<0.001** | 0.62 | 0.810 | **-3.03** | **0.007** |
| F | Day | Summer | Winter | **-5.61** | **<0.001** | 0.66 | 0.788 | **-3.01** | **0.007** |
| G | Day | Temperate | Summer | **3.18** | **0.004** | -0.72 | 0.754 | 1.92 | 0.132 |
| G | Day | Temperate | Winter | -1.72 | 0.198 | -0.43 | 0.902 | -0.92 | 0.628 |
| G | Day | Summer | Winter | **-4.93** | **<0.001** | 0.27 | 0.959 | **-2.86** | **0.012** |
| H | Day | Temperate | Summer | **8.52** | **<0.001** | **3.54** | **0.001** | 1.16 | 0.478 |
| H | Day | Temperate | Winter | -0.88 | 0.654 | **3.18** | **0.004** | **-2.83** | **0.013** |
| H | Day | Summer | Winter | **-7.85** | **<0.001** | 0.16 | 0.986 | **-3.71** | **0.001** |
| C | Day | Temperate | Summer | -0.03 | 1.000 | **2.58** | **0.027** | -0.92 | 0.629 |
| C | Day | Temperate | Winter | **-4.07** | **<0.001** | 1.93 | 0.131 | **-2.67** | **0.021** |
| C | Day | Summer | Winter | **-4.06** | **<0.001** | 0.70 | 0.765 | -2.06 | 0.098 |
| B | Day | Temperate | Summer | **6.96** | **<0.001** | 1.73 | 0.193 | **2.99** | **0.008** |
| B | Day | Temperate | Winter | **-3.99** | **<0.001** | 2.11 | 0.087 | **-4.53** | **<0.001** |
| B | Day | Summer | Winter | **-10.41** | **<0.001** | 0.52 | 0.863 | **-7.31** | **<0.001** |
| E | Day | Temperate | Summer | **3.98** | **<0.001** | -0.36 | 0.930 | **3.78** | **<0.001** |
| E | Day | Temperate | Winter | 0.48 | 0.879 | **-4.93** | **<0.001** | 1.75 | 0.185 |
| E | Day | Summer | Winter | **-2.63** | **0.023** | **-4.81** | **<0.001** | -1.34 | 0.371 |
| A | Day | Temperate | Summer | 0.31 | 0.947 | **2.49** | **0.034** | -0.94 | 0.617 |
| A | Day | Temperate | Winter | -1.91 | 0.135 | **2.68** | **0.020** | **-2.36** | **0.048** |
| A | Day | Summer | Winter | **-2.41** | **0.043** | 0.60 | 0.820 | -1.70 | 0.206 |
| D | Day | Temperate | Summer | -0.84 | 0.677 | 0.10 | 0.994 | -0.23 | 0.970 |
| D | Day | Temperate | Winter | -2.25 | 0.064 | 1.19 | 0.459 | -1.67 | 0.218 |

| | | | | | | | | | |
|---|---|---|---|---|---|---|---|---|---|
| D | Day | Summer | Winter | -1.59 | 0.251 | 1.10 | 0.512 | -1.46 | 0.309 |
