# Supplementary material for: Dairy Cow Behavior Is Affected by Period, Time of Day and Housing
Source: Animals (Basel). 2022 Feb 18;12(4):512. doi: 10.3390/ani12040512 (PMC8868199; doi:10.3390/ani12040512)
Supplement: Supplementary file 1 [file animals-12-00512-s001.zip › Leliveld_TableS3.pdf]

Table S3: Means of daily values per farm and per period for lying duration (LD), number of lying bouts (NLB) and mean lying bout duration (MLBD). * The units for LD and NLB are different than those used for daytime and nighttime values.

| **Parameter** | **Period** | **Farm** | | | | | | | |
|---|---|---|---|---|---|---|---|---|---|
| | | **A** | **B** | **C** | **D** | **E** | **F** | **G** | **H** |
| LD (hr)* | winter | 12.00 | 12.81 | 12.47 | 11.21 | 10.94 | 13.59 | 12.03 | 11.66 |
| | temperate | 9.94 | 9.53 | 9.71 | 11.49 | 9.40 | 9.45 | 11.68 | 13.47 |
| | summer | 9.56 | 7.98 | 10.49 | 12.42 | 9.00 | 10.67 | 9.94 | 10.30 |
| NLB (n)* | winter | 10.92 | 7.87 | 13.70 | 9.98 | 10.28 | 10.40 | 8.78 | 9.42 |
| | temperate | 11.20 | 9.22 | 17.28 | 12.87 | 5.78 | 10.12 | 8.60 | 10.63 |
| | summer | 10.20 | 8.63 | 15.80 | 12.45 | 6.97 | 10.27 | 8.98 | 9.62 |
| MLBD (min) | winter | 69.91 | 101.80 | 58.48 | 72.39 | 91.76 | 83.02 | 89.10 | 79.90 |
| | temperate | 57.43 | 69.33 | 34.78 | 58.98 | 108.76 | 63.66 | 84.23 | 80.54 |
| | summer | 60.27 | 59.94 | 42.35 | 63.71 | 85.07 | 65.78 | 77.64 | 68.86 |
